# Growth-inducing effects of argon plasma on soybean sprouts via the regulation of demethylation levels of energy metabolism-related genes

**DOI:** 10.1038/srep41917

**Published:** 2017-02-07

**Authors:** Jiao Jiao Zhang, Jin Oh Jo, Do Luong Huynh, Raj Kumar Mongre, Mrinmoy Ghosh, Amit Kumar Singh, Sang Baek Lee, Young Sun Mok, Park Hyuk, Dong Kee Jeong

**Affiliations:** 1 Laboratory of Animal Genetic Engineering and Stem Cell Biology, Department of Animal Biotechnology and Advance Next Generation Convergence Technology, Jeju National University, Jeju 690756, South Korea; 2 Department of Chemical and Biological Engineering, Jeju National University, Jeju 690756, South Korea; 3 Intellectual Property Law Firm PCR, Seoul 06194, South Korea

## Abstract

This study was conducted to determine the effects of argon plasma on the growth of soybean [*Glycine max* (L.) Merr.] sprouts and investigate the regulation mechanism of energy metabolism. The germination and growth characteristics were modified by argon plasma at different potentials and exposure durations. Upon investigation, plasma treatment at 22.1 kV for 12 s maximized the germination and seedling growth of soybean, increasing the concentrations of soluble protein, antioxidant enzymes, and adenosine triphosphate (ATP) as well as up-regulating *ATP a1, ATP a2, ATP b1, ATP b2, ATP b3*, target of rapamycin (*TOR*), growth-regulating factor (*GRF) 1–6*, down-regulating *ATP MI25* mRNA expression, and increasing the demethylation levels of the sequenced region of *ATP a1, ATP b1, TOR, GRF 5*, and *GRF 6* of 6-day-old soybean sprouts. These observations indicate that argon plasma promotes soybean seed germination and sprout growth by regulating the demethylation levels of *ATP, TOR*, and *GRF*.

Plasma is an electrically neutral medium consisting of positive and negative ions, free electrons, excited molecules, and radicals. High potential of plasma may elevate mutation rate as it is accompanied by an increased concentration of charged ions; however, there is little physiological damage to seeds that are subjected to such plasma^1^,^2^,^3^. Plasma seed treatment is an eco-agricultural technology which may stimulate plant growth. Currently, the main area of research in plasma biological engineering is cultivation of plasma-activated seeds to improve the quality and production of crops^4^,^5^,^6^,^7^,^8^. Soybean sprouts are one of the most common and economic vegetables in East Asian countries owing to its high nutritional value and simple production method. It typically takes 1 week to reach maximum growth. Shortening growth period and increasing quality and yield of soybean sprouts are becoming issues which farmers are concerned most about. Our research explores the possibility of argon plasma promoting soybean seed germination and sprout growth.

Radiation from different types of plasma stimulated seed growth, increased germination and resistance to adversity, and improved the quality and yield of crops^2^,^5^,^7^,^9^. Our study is the first to investigate the application of argon plasma on soybean sprout growth. Different potentials and exposure durations of plasma show various effects on plant growth. Short-duration exposure of low-temperature plasma significantly increases the length and weight of roots, whereas increasing the exposure duration inhibits them^10^. Dhayala *et al*. found that the effects of a short-duration low-pressure plasma treatment on safflower seed germination were much more effective than those of a long-duration high-pressure plasma treatment^1^. The effect of plasma treatment with different intensities (output power) was different^5^. Our objective is to find the efficacious exposure potential and duration of argon plasma on soybean sprout growth.

How plasma affects seed germination and growth is not well understood. Magnetized arc plasma promotes tomato seed germination and plant growth by activating various enzymes and increasing biological chemical energy via high-pressure arc plasma electromagnetic effect of active particles^11^,^12^. A cold plasma-reacted deposit on the surface of seeds would either delay or accelerate seed germination by modifying seed coats^2^. Sera *et al*. reported that plasma can generate UV radiation, radicals, and chemical reactions, which play an important role in dormancy breaking early, resulting in the acceleration of seed germination^8^,^13^,^14^. Helium plasma was exploited to promote faster germination by improving the wettability and water imbibition of seeds through reaction with electrons, ions, and radicals emitted in the plasma discharge^4^,^5^. Ion exchange capacity enhances enzyme transformation speed and increases the concentrations of soluble sugar and soluble protein, thereby improving germination rate of plasma-treated rice seeds^10^. However, there are no current studies exploring gene regulation mechanism of plasma on seed and plant growth.

Cytosine methylation is a fundamental epigenetic mechanism in the regulation of gene expression and transposable elements in plants^15^. CG, CHG, and CHH types (where H is A, C or T) have been observed in plant genomes^16^. The differences in methylation pattern observed at certain regulatory sites may be involved in the stress responsive mechanism in plants^15^. Extensive methylation of CpG islands (‘p’ designates the phosphodiester bond that joins two nucleotides), which are located in the GC-rich regions of promoters and end regions of transcribed portions^17^, is associated with transcriptional inactivation^16^,^18^ and regulates gene expression across plant development and during durations of external stress^19^,^20^,^21^. Demethylation of promoters is required for the uni-parental expression of imprinting genes in the endosperm, which is essential for seed viability^22^.

Therefore, questions regarding whether argon plasma has effects on soybean sprout growth and how to regulate the energy metabolism of seedling through cytosine demethylation remain unanswered.

## Results

### Soybean germination and sprout growth

Soybean sprout growth improved within a certain range of plasma potential (10.8–22.1 kV) at 1 min exposure (see Supplementary Fig. S1). However, at 2 min of exposure duration, maximum soybean sprout growth was obtained at 17.3 kV, but decreased with further increase in plasma potential till growth is eventually inhibited at >21.2 kV (see Supplementary Fig. S2). The ge6rmination and production rates of soybean sprouts exposed to plasma at 22.1 kV for 1 min showed the highest increases compared with those of the control group, but longer plasma exposure duration of 2 min had significant contrary effects (see Supplementary Table S3).

**Figure 1:**
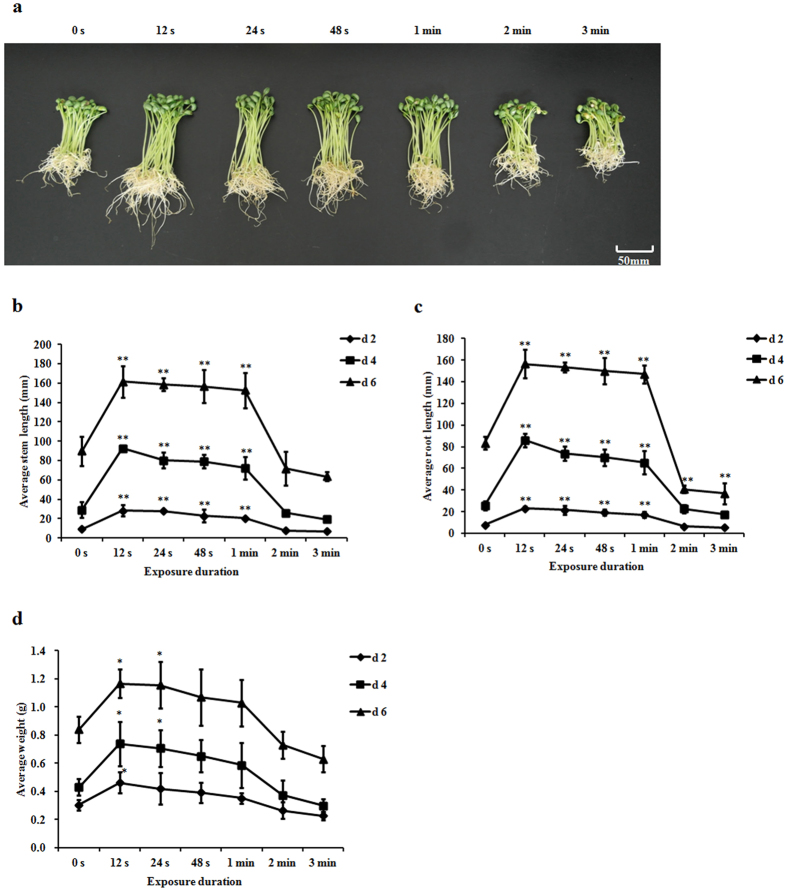
Effects of plasma at 22.1 kV for different exposure duration on (**a**) 6-day-old soybean sprouts appearance, (**b**) average stem length, (**c**) average root length, and (**d**) average weight of soybean sprouts on days 2, 4, and 6. Error bars indicated standard error (n = 3). *p < 0.05 versus control; **p < 0.01 versus control, according to LSD test.

The weight of soybean sprout and the lengths of stem and root increased within the exposure duration range of 12 s–1 min, peaking at 12 s and 22.1 kV on days 2, 4, and 6 (Fig. 1). In detail, the average stem and root lengths of soybean sprouts in plasma-treated group at 22.1 kV for 12 s were 161.45 and 156.45 mm on day 6, with 0.79- (*p* < 0.001) and 0.88-fold increases (*p* < 0.001), respectively, compared with those of the control group (Fig. 1b and c). In addition, soybean sprouts exposed to plasma at 22.1 kV for 12 s exhibited a 0.39-fold increase in weight (*p* = 0.033) on day 6 compared with that of the control group (Fig. 1d). However, longer exposure duration (>2 min) at 22.1 kV inhibited their growth. Similarly, the germination and production rates of soybean sprouts in plasma-treated group at 22.1 kV for 12 s showed the greatest increases of 0.15- (*p* < 0.001) and 0.67-fold (*p* < 0.001), respectively, compared with those of the control group (Table 1).

### Soluble protein, antioxidant enzymes, and adenosine triphosphate

**Figure 2:**
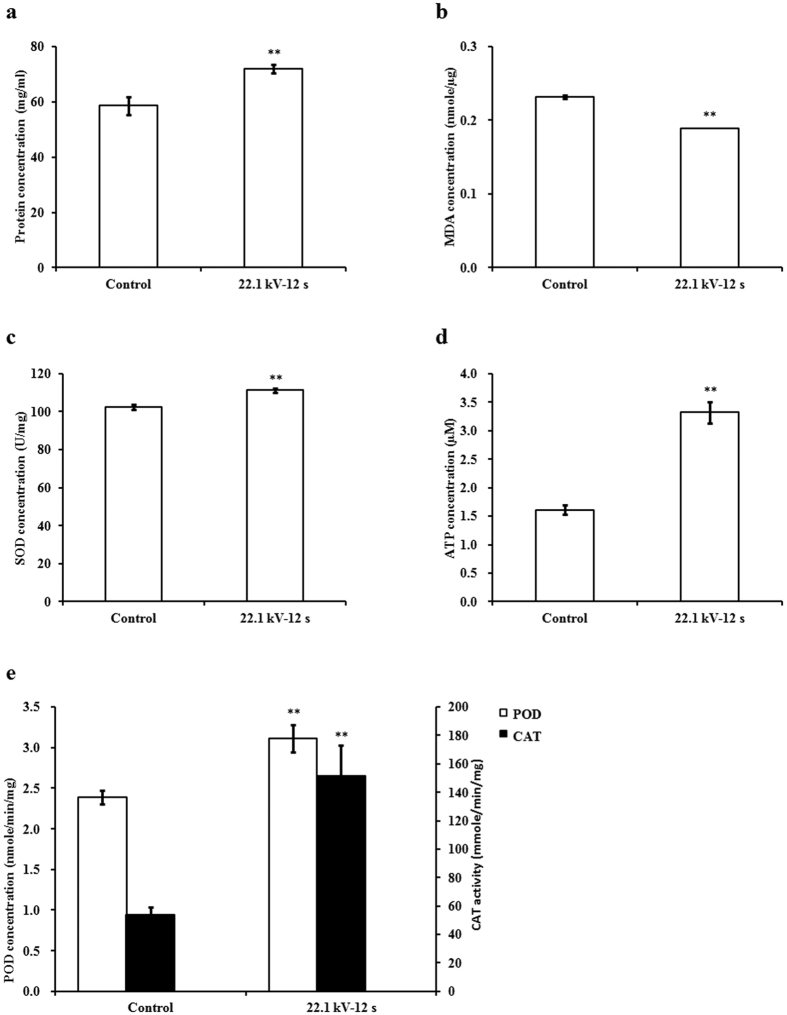
Effects of plasma on concentrations of (**a**) soluble protein, (**b**) malondialdehyde (MDA), (**c**) superoxide dismutase (SOD), (**d**) adenosine triphosphate (ATP), and (**e**) peroxidase (POD) and catalase (CAT) of 6-day-old soybean sprouts. Error bars indicated standard error (n = 3). *p < 0.05 versus control; **p < 0.01 versus control, according to LSD test.

Six-day-old soybean sprouts in plasma-treated group at 22.1 kV for 12 s showed significant increases in concentrations of soluble protein, superoxide dismutase (SOD), peroxidase (POD), catalase (CAT), and adenosine triphosphate (ATP) by 0.23- (*p* = 0.007), 0.08- (*p* = 0.002), 0.31- (*p* = 0.005), 1.82- (*p* = 0.003), and 1.06-fold (*p* = 0.001), respectively, but significant decrease in malondialdehyde (MDA) concentration by 0.18-fold (*p* < 0.001) compared with those of the control group (Fig. 2).

### Ethanol concentration in anaerobic respiration process

**Figure 3:**
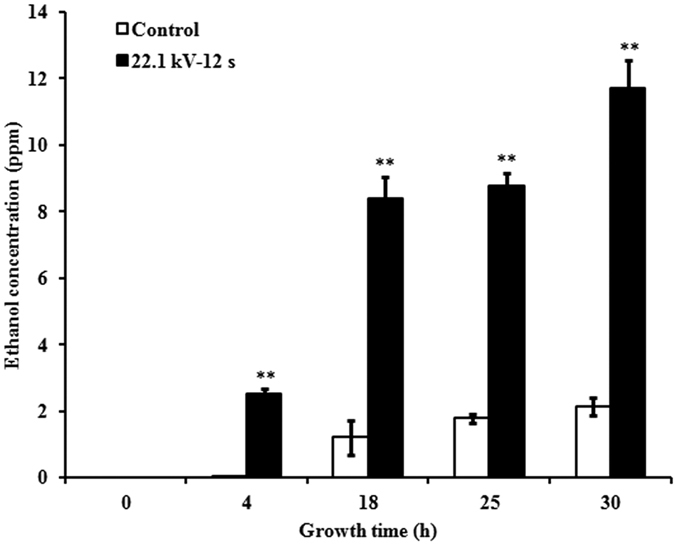
Ethanol concentration in anaerobic respiration process. Ethanol concentration was measured after 0, 4, 18, 25, and 30 h air proof using a gas chromatograph. Error bars indicated standard error (n = 3). *p < 0.05 versus control; **p < 0.01 versus control, according to LSD test.

In a sealed environment, ethanol concentrations produced by soybean sprouts increased in control and plasma-treated groups at 22.1 kV for 12 s with their growth period was extended. Notably, ethanol concentration in the plasma-treated group increased at a much higher rate than that in the control group, particularly between 0 h and 4 h (2.50 and 0.03 ppm in the plasma-treated and control groups, respectively). Compared with the control group, ethanol concentration in the plasma-treated group significantly increased during the growth period, showing 5.91- (*p* < 0.001), 3.93- (*p* < 0.001), and 4.46-fold (*p* < 0.001) increases at 18, 25, and 30 h, respectively (Fig. 3).

### mRNA expressions of ATPs, target of rapamycin, and growth-regulating factors

**Figure 4:**
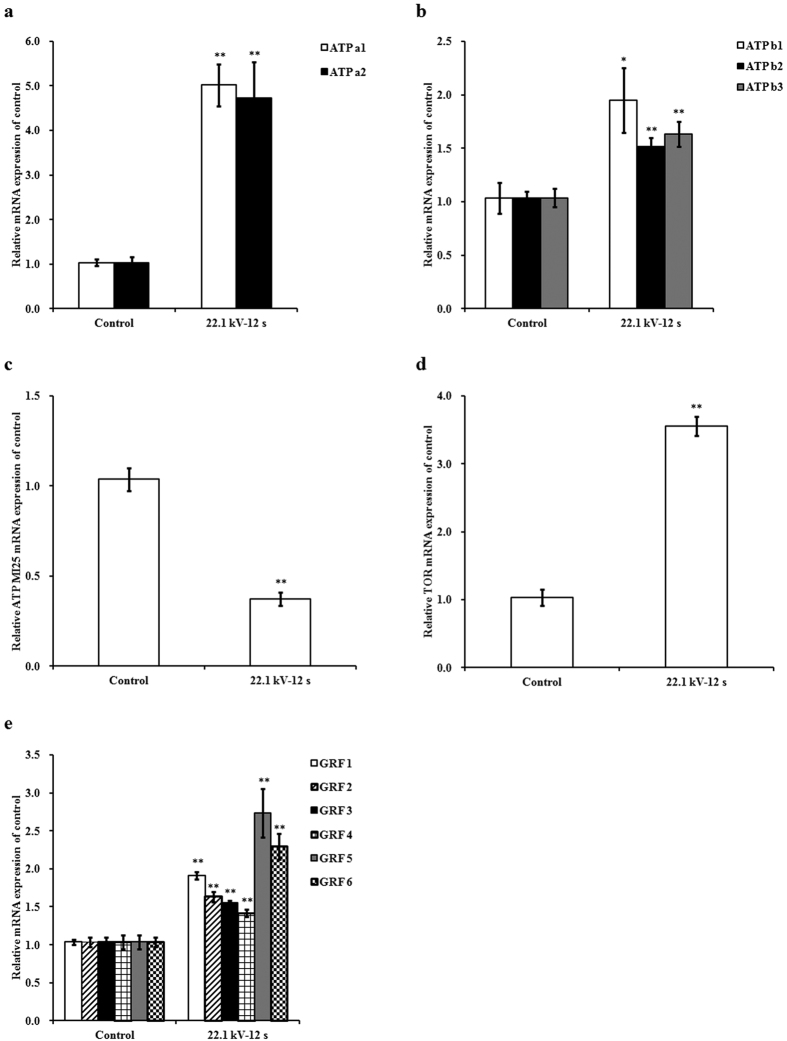
Effects of plasma on mRNA expressions of (**a**) *ATP a1* and *ATP a2*, (**b**) *ATP b1, ATP b2*, and *ATP b3*, (**c**) *ATP MI25*, (**d**) target of rapamycin (*TOR*), and (**e**) growth-regulating factor (*GRF) 1–6* in soybean sprouts on day 6. mRNA levels were determined using real-time RT-PCR, and expressed relative to those of *β-actin*. Error bars indicated standard error (n = 3). *p < 0.05 versus control; **p < 0.01 versus control, according to LSD test.

The expressions of *ATP a1* [*Glycine max* ATP synthase subunit alpha, chloroplastic-like (LOC100815582)] and *ATP a2* [*G. max* ATP synthase subunit alpha, chloroplastic-like (LOC102661828)] mRNAs of the 6-day-old soybean sprouts in plasma-treated group at 22.1 kV for 12 s increased by 3.84- (*p* < 0.001) and 3.56-fold (*p* = 0.003), respectively, compared with those of the control group (Fig. 4a). Increases in the mRNA expressions of *ATP b1* [*G. max* ATP synthase subunit beta, mitochondrial-like (LOC100797948)], *ATP b2* [*G. max* ATP synthase subunit beta, mitochondrial-like (LOC100789705)], and *ATP b3* [*G. max* ATP synthase subunit beta, mitochondrial-like (LOC100783395)] in the plasma-treated group were less than those in *ATP a1* and *ATP a2* mRNAs expressions of the plasma-treated group. Exposure to plasma at 22.1 kV for 12 s significantly increased the mRNA expressions of *ATP b1, ATP b2*, and *ATP b3* by 0.88- (*p* = 0.018), 0.46- (*p* = 0.003), and 0.58-fold (*p* = 0.004), respectively, compared with those of the control group (Fig. 4b).

Exposures to plasma at 22.1 kV for 12 s significantly decreased the expression of *ATP MI25* [*G. max* ATP synthase protein MI25-like (LOC100816723)] mRNA by 0.64-fold (*p* < 0.001) (Fig. 4c) and increased the mRNA expression of target of rapamycin (*TOR*) in 6-day-old soybean sprouts by 2.43-fold (*p* < 0.001) (Fig. 4d) compared with those of the control group. Argon plasma enhanced mRNA expressions of growth-regulating factor (*GRF) 1–6* in soybean sprouts on day 6. Exposure to plasma at 22.1 kV for 12 s increased the mRNA expressions of *GRF 1, GRF 2, GRF 3, GRF 4, GRF 5*, and *GRF 6* by 0.84- (*p* < 0.001), 0.58- (*p* = 0.001), 0.50- (*p* = 0.001), 0.36- (*p* = 0.007), 1.64- (*p* = 0.002), and 1.21-fold (*p* = 0.001), respectively, compared with those of the control group (Fig. 4e).

### DNA methylation

**Figure 5:**
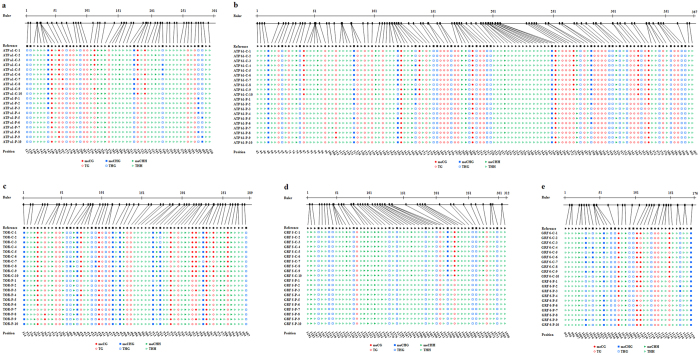
Cytosine methylation analysis results of (**a**) *ATP a1*, (**b**) *ATP b1*, (**c**) *TOR*, (**d**) *GRF 5*, and (**e**) *GRF 6* of 6-day-old soybean sprouts were analyzed using CyMATE. The length of sequenced region and position of cytosine are shown schematically. The order of the individual sequences of ten clones is listed on the left. C represents the control group, P represents plasma-treated group at 22.1 kV for 12 s. Reference sequence is shown in the first line. The sequence is distinguished by circles for CG, squares for CHG, and triangles for CHH. Filled symbols represent methylated cytosine, open symbols represent unmethylated cytosine.

Bisulfite sequencing of *ATP a1, ATP b1, TOR, GRF 5*, and *GRF 6* of the 6-day-old soybean sprouts was performed to find out the exact location, type, and extent of methylation. The sequence analysis results of these five genes using CyMATE software were shown in Fig. 5. The methylation levels in the sequenced region of *ATP a1* (35/520, 6.73% methylation ratio), *ATP b1* (111/1220, 9.10%), *TOR* (181/630, 28.73%), *GRF 5* (30/560, 5.36%), and *GRF 6* (68/360, 18.89%) in plasma-treated group at 22.1 kV for 12 s decreased by 0.12-, 0.07-, 0.11-, 0.13-, and 0.06-fold, respectively, compared with those of the control group (*ATP a1*, 95/520, 18.27%; *ATP b1*, 201/1220, 16.48%; *TOR*, 253/630, 40.16%; *GRF 5*, 102/560, 18.21%; *GRF 6*, 89/360, 24.72%) (Fig. 5). The decrease of average methylation levels in the sequenced region of *ATP a1, ATP b1, TOR, GRF 5*, and *GRF 6* for CG type was found to be more than CHG and CHH after plasma exposure.

**Figure 6:**
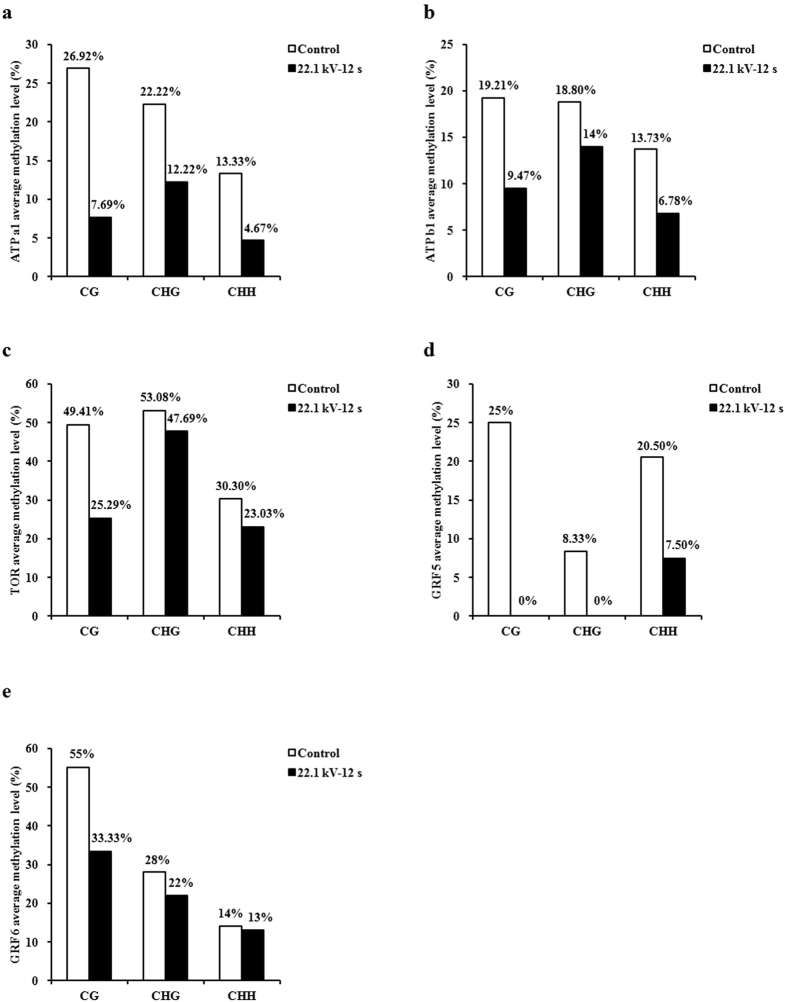
Average methylation level for CG, CHG, and CHH within the sequenced region of (**a**) *ATP a1*, (**b**) *ATP b1*, (**c**) *TOR*, (**d**) *GRF 5*, and (**e**) *GRF 6* of 6-day-old soybean sprouts.

In detail, average methylation level in *ATP a1* for CG (7.69%), CHG (12.22%), and CHH (4.67%) in plasma-treated group decreased by 0.19-, 0.10-, and 0.09-fold, respectively, compared with those of the control group (CG, 26.92%; CHG, 22.22%; CHH, 13.33%) (Fig. 6a). Average methylation level in *ATP b1* for CG (9.47%), CHG (14%), and CHH (6.78%) in plasma-treated group decreased by 0.10-, 0.05-, and 0.07-fold, respectively, compared with those of the control group (CG, 19.21%; CHG, 18.80%; CHH, 13.73%) (Fig. 6b). Average methylation level in *TOR* for CG (25.29%), CHG (47.69%), and CHH (23.03%) in plasma-treated group decreased by 0.24-, 0.05-, and 0.07-fold, respectively, compared with those of the control group (CG, 49.41%; CHG, 53.08%; CHH, 30.30%) (Fig. 6c). No methylation in *GRF 5* for CG and CHG was found in plasma-treated group (Fig. 6d), average methylation level for CG and CHG was 25% and 8.33% in the control group, respectively. Average methylation level in *GRF 5* for CHH was 7.50% in plasma-treated group, decreased by 0.13-fold compared with that of the control group (20.50%). Average methylation level in *GRF 6* for CG (33.33%), CHG (22%), and CHH (13%) in plasma-treated group decreased by 0.22-, 0.06-, and 0.01-fold, respectively, compared with those of the control group (CG, 55%; CHG, 28%; CHH, 14%) (Fig. 6e).

## Discussion

This study showed that an appropriate exposure potential and duration of argon plasma promoted soybean seed germination and sprout growth. Li Ling *et al*. reported that cold plasma increased the germination and vigor indices of soybean seeds, but lower and higher energy levels had no significant influence on seed germination^23^. Short-duration exposure of low-temperature plasma significantly increased the length and weight of roots, but longer-duration exposure had an inhibitive effect on these parameters^10^. Dhayala *et al*. found that a short-duration low-pressure plasma treatment on safflower seed germination was much more effective than a long-duration high-pressure plasma treatment^1^. The effect of plasma treatment with different output power was different^5^. In this study, soybeans exposed to plasma at 22.1 kV for 12 s showed considerable improvement in terms of seedling growth compared with those exposed to plasma at lower potentials and longer durations.

Soluble proteins reflect the overall metabolism of plants^24^,^25^,^26^. Increase and accumulation of soluble proteins improve water retention capacity and imbibition of cells^4^,^6^,^23^. This may account for the promotion of soybean seed germination and growth as plasma treatment helps improve water uptake^2^,^6^. MDA reflects the extent of lipid peroxidation and radical attack and upregulates genes implicated in biotic stress response in plants^27^,^28^. SOD acts as an antioxidant and protects the plant cell from being oxidized by reactive oxygen species (ROS), which are involved in plant metabolic activity^29^. POD and CAT can catalyze the decomposition of hydrogen peroxide (H_2_O_2_) and reduce the deleterious effects of H_2_O_2_ on organelles during stress responses in plants^30^. Yin *et al*. found that the arc discharge plasma pretreatment of tomato seeds increased the activities of CAT, SOD, and POD, and markedly improved active oxygen metabolism level and ATP level, resulting in the acceleration of seed germination^11^,^12^. In our study, argon plasma reduced MDA concentration and increased the activities of SOD, POD, and CAT in soybean sprouts, indicating that plasma exposure activated the antioxidant system and increased the resistance of soybean sprouts from the stress response, which may indirectly contribute to the promotion of germination and metabolism.

Increase in ATP concentration in seeds promotes the germination and growth of sprouts^11^,^12^,^31^. We found that plasma-treated soybean sprouts showed increases in ATP concentration in an aerobic environment; they also showed more ethanol production, which indicates more ATP production during anaerobic respiration. The mRNA expressions of ATP synthase subunit alpha genes in the chloroplasts showed greater increase than those of the subunit beta genes in mitochondria, which indicated that argon plasma may mainly target the ATP synthase subunit alpha genes in the chloroplasts of soybean sprouts. Cytosine methylation is associated with alteration of gene transcription, leading to morphological changes without changing the sequence^32^. The changes in the initial action point of external factors on the transcription factor-binding target sites has a direct impact on the expression of the concerned genes and, subsequently, on phenotypic characters^15^. This could be explained by the fact that hypermethylation alters the chromatin structure, preventing normal interaction of the DNA strand with the transcriptional machinery or that hypomethylation activates some previously silenced genes^33^. Demethylation of CpG islands has been associated with transcriptional activation of selected imprinted genes^34^. DNA methylation sequencing results obtained in the sequenced region of soybean sprouts showed that decrease in the methylation level after plasma exposure of *ATP a1* (60/520, 11.54%) was higher than that of *ATP b1* (90/1220, 7.38%), which may explain why mRNA expression levels of ATP synthase subunit alpha genes in the chloroplasts were higher than those of subunit beta genes in the mitochondria.

ATP MI25, one of the chains of the non-enzymatic component of the mitochondrial ATPase complex, has activities similar to those of the hydrogen ion transmembrane transporter and uses ATP hydrolysis to drive the transport of ATP synthesis-coupled proton across the membrane based on the GO annotation of *ATP MI25*. Our study found that argon plasma decreased the *ATP MI25* mRNA expression, which indicated that argon plasma reduced the ATP hydrolysis of soybean sprout mitochondria and inhibited the transmembrane transport of hydrogen ion and ATP synthesis-coupled proton. This may be caused by the accumulation of synthetic polymers induced by plasma on the surface of soybean, resulting in a decrease in the apparent contact angle^35^. Bormashenko *et al*. did not observe restored hydrophobicity in seeds exposed to cold plasma because of the changes in the physical and chemical properties of lentil beans^36^.

TOR orchestrates transcriptional networks that wire central metabolism and biosynthesis for energy and biomass production. In addition, these networks integrate localized stem/progenitor cell proliferation through inter-organ nutrient coordination to control developmental transitions and growth^37^. Montane *et al*. analyzed TOR inhibitors, which were ATP competitive as they targeted the ATP-binding pocket of the kinase domain and inhibited the early differentiation of meristematic cells and root growth of *Arabidopsis thaliana* and diverse angiosperms^38^. Moderate increases in TOR expression levels (<2-fold) have been found to increase root and shoot growth, cell size, and seed yield without visibly affecting plant morphology^39^. In our study, the methylation level in the sequenced region of *TOR* showed a significant decrease after plasma exposure (72/630, 11.43%); this was associated with transcriptional activation of *TOR*. Argon plasma exposure increased the expression of *TOR* mRNA in soybean sprouts. As a result, the improved TOR kinase activity may induce increases in the metabolism and biosynthesis for energy and biomass production, resulting in promotion of stem and root growth of soybean sprouts. Rapamycin (TOR inhibitor) or the estradiol-inducible TOR mutant completely abolishes glucose-promoting root hair growth associated with ROS signaling^40^,^41^, which might also explain how the argon plasma participated in the modulation of antioxidant enzymes in our study. Therefore, we inferred that *TOR* demethylation level induced the promotion of soybean sprout growth associated with ROS signaling.

GRFs are plant-specific proteins that play important roles in regulating plant growth and development^42^. GRF genes have been reported to act as positive regulators of leaf size through the promotion and/or maintenance of cell proliferation activity in leaf primordia^43^,^44^,^45^. Our study found that argon plasma exposure enhanced the mRNA expressions of *GRF 1, GRF 2, GRF 3, GRF 4, GRF 5*, and *GRF 6* in soybean sprouts, and this may promote cell proliferation activity in the cotyledon of soybean sprouts. *GRF 5* and *GRF 6* showed higher mRNA expressions than other GRF genes. Cytosine methylation analysis results obtained in the sequenced region of soybean sprouts showed that decrease in the methylation level after plasma exposure of *GRF 5* (72/560, 12.86%) was higher than that of *GRF 6* (21/360, 5.83%), which may explain why mRNA expression level of *GRF 5* was higher than that of *GRF 6*. Interestingly, no methylation in *GRF 5* for CG and CHG within the sequenced region was found after plasma exposure, pointing out argon plasma had a complete demethylation effect on *GRF 5* for CG and CHG types of soybean sprouts. The differences in methylation pattern observed at certain regulatory sites indicate the direct impact of stress on genome and site-specific stress-induced DNA methylation^15^. A noticeable phenomenon was the decrease of average methylation levels in the sequenced region of *ATP a1, ATP b1, TOR, GRF 5*, and *GRF 6* for CG type was more than CHG and CHH after plasma exposure. It is very likely that CG type may be a prominent demethylation pattern of soybean sprouts exposed to plasma at 22.1 kV for 12 s.

In summary, our study suggests argon plasma promotes soybean seed germination and sprout growth by increasing the concentrations of soluble protein and antioxidant enzymes and regulating the demethylation levels of *ATP, TOR*, and *GRF*. Argon plasma exposure technology provides a more efficient and viable approach toward soybean sprouts growth promotion and improves resistance to hypoxic environments.

## Methods

### Plasma treatment

**Figure 7:**
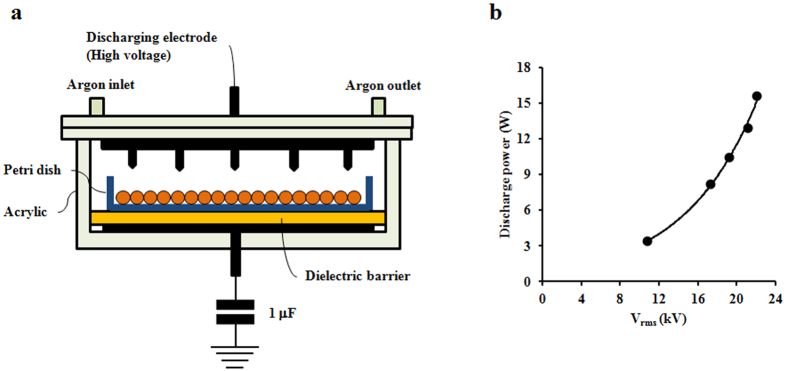
Plasma treatment. (**a**) Plasma reactor schematic. (**b**) Voltage and discharge power applied in plasma treatment.

As shown in Fig. 7a, the plasma reactor is based on dielectric barrier discharge operating at atmospheric pressure. The plasma reactor consists of two 100 mm wide disk-shaped electrodes and a 5-mm thick glass dielectric barrier. The upper electrode has 16 needles (thickness: 1 mm; length: 2.5 mm) that are evenly distributed on its inner surface, pointing downward to the lower electrode. The distance between the needle tip and the ceramic dielectric barrier placed on the lower electrode is 30 mm. The plasma reactor is energized by an alternating current high voltage (operating frequency: 60 Hz) to create plasma. Pure argon is fed to the plasma reactor at a flow rate of 2 l/min. The voltage applied to the plasma reactor is measured using a digital oscilloscope (Tektronix, Beaverton, OR, USA) and a 1000:1 high voltage probe (P6015, Tektronix). The discharge power is determined using a voltage–charge Lissajous plot, where the charge is recorded by measuring the voltage across the 1.0-μF capacitor connected to the plasma reactor in series. As shown in Fig. 7b, the discharge power increases exponentially from 3.4 to 15.6 W, as the voltage is changed from 10.8 to 22.1 kV rms.

### Growth situation assay

The experiment was conducted at the Department of Animal Biotechnology, Advance Next Generation Convergence Technology, and Chemical and Biological Engineering, Jeju National University, Jeju, South Korea. Full and intact yellow soybean seeds (one seed weighed approximately 0.13 g, and there were 20 seeds in each group), purchased in Jeju, South Korea, were exposed to plasma at different potentials and durations at 25 °C. Soybean seeds without plasma treatment were used as the control group. Seeds were soaked in 10 ml distilled water for 6 h before placing them in the bean sprout machine with 1 l distilled water inside, and they were incubated for 6 days at 25 °C and 100% relative humidity. The germination rate was recorded when the germ length was more than half of the seed length. The total weight of a soybean sprout and the lengths of stems and roots were measured on days 2, 4, and 6. The experiment was designed as completely randomized with three replications.

1. Germination rate (%) = (Number of germinated soybean seeds in 6 days/total number of soybean seeds) × 100%.
2. Production rate (%) = Soybean sprout stem weight (g) on day 6 × germination rate (%)/soybean seed weight (g).

### Soluble protein, antioxidant enzymes, and ATP analysis

Soybean seeds without plasma treatment (control) and those treated with 22.1 kV for 12 s were grown for 6 days. Homogenization of soybean sprout was performed in 1 ml ice-cold phosphate buffer saline (0.05 mol/l, pH 7.4), centrifuged (12,000 × *g*) for 10 min at 4 °C, and supernatant was collected to detect soluble protein concentration using a bicinchoninic acid protein assay kit (Sigma-Aldrich, St. Louis, MO, USA). Activities of MDA, SOD, POD, and CAT were analyzed using kits from Sigma-Aldrich, according to the manufacturer’s instructions. On day 6, soybean sprouts were collected and homogenized in 1× ATP reaction buffer. After centrifugation (12,000 × *g*, 5 min, 4 °C), ATP concentration in the supernatant was determined using an ATP determination kit (Invitrogen, Carlsbad, CA, USA). Relative light unit values were measured using a luminometer (Sirius L Tube Luminometer, Titertek Berthold, Bleichstraße, Pforzheim, Germany).

### Gas chromatography

Soybean seeds without plasma treatment (control) and those treated with 22.1 kV for 12 s were grown for 3 days in the bean sprout machine, which was shielded and completely covered. Soybean sprouts were then collected after 0, 4, 18, 25, and 30 h air proof. The change in concentration of gaseous hydrocarbon produced during growth was monitored using a gas chromatograph (Bruker 450-GC, Billerica, MA, USA) equipped with a flame ionization detector. The conditions of gas chromatography were as follows: oven temperature: 50–59 °C (1 °C/min); Column: BR-624 ms (60 m, 0.32 mm, 1.80 μm, Bruker); Column flow: 2 ml/min; Injector temperature: 150 °C; Detector temperature: 270 °C.

### Real-time RT-PCR analysis

Total RNA was extracted and purified from the control and plasma-treated groups using the TRI Reagent RT (Invitrogen), following the manufacturer’s instructions. RT-PCR analysis of mRNAs expression was performed using SuperScript III First-Strand Synthesis System (Invitrogen), Prime Taq DNA Polymerase (GENETBIO, Daejeon, South Korea), EvaGreen Dye (Biotium, Hayward, CA, USA). First-strand cDNA synthesis was performed using 1 μl total RNA (1 μg/μl), 1 μl 50 μM oligo(dT)_20_, 1 μl 10 mM dNTP mix, and 7 μl DEPC-treated water and was incubated at 65 °C for 5 min and put on ice for 2 min, then again incubated at 50 °C for 50 min with 2 μl 10× RT buffer, 4 μl 25 mM MgCl_2_, 2 μl 0.1 M DTT, 1 μl RNaseOUT (40 U/μl), 1 μl SuperScript III RT (200 U/μl), terminated at 85 °C for 5 min. RT-PCR was performed using 2.5 μl 10× reaction buffer, 2 μl 10 mM dNTPs Mixture, 1 μl Upstream Primer (5 pmole/μl), 1 μl Downstream Primer (5 pmole/μl), 0.2 μl Prime Taq DNA Polymerase, 1 μl cDNA, 1.25 μl EvaGreen Dye, 16.05 μl DEPC-treated water using a StepOne real-time PCR system (Applied Biosystems, Waltham, MA, USA). RT-PCR conditions included initial denaturation at 95 °C for 10 min, denaturation at 95 °C for 15 s, annealing at 60 °C (*β-actin, ATP a1, ATP a2, ATP b1, ATP b2, ATP b3, ATP MI25, TOR, GRF 3, GRF 4*, and *GRF 6*) or 62 °C (*GRF 1, GRF 2*, and *GRF 5*) for 30 s (primers see Supplementary Table S4). Forty cycles were conducted, and melt curve was performed under 95 °C for 15 s, 60 °C for 1 min, and increased to 95 °C for 15 s by 0.3 °C.

The cycle threshold value of each sample was determined using triplicate measurements. The equivalent dilution was calculated according to the standard curve and then normalized to the housekeeping gene (*β-actin*). RT-PCR reaction product sequences were confirmed by direct nucleotide sequencing using an ABI PRISM 7700 Sequence Detector (Applied Biosystems). Relative expression levels were calculated using the 2^−ΔΔCT^ method^46^.

### Bisulfite conversion and bisulfite-sequencing PCR

Genomic DNA was extracted and purified from 6-day-old soybean sprouts using cetyl trimethylammonium bromide method^47^. Sodium bisulfite conversion was performed using 1 μl genomic DNA (1 μg/μl), 19 μl RNase-free water, 85 μl Bisulfite mix, and 35 μl DNA protect buffer with a Master Cycler Gradient (Eppendorf, Hamburg, Germany). The bisulfite conversion conditions were as follows: denaturation at 95 °C for 5 min, incubation at 60 °C for 25 min, denaturation at 95 °C for 5 min, incubation at 60 °C for 85 min, denaturation at 95 °C for 5 min, and incubation at 60 °C for 175 min, following to the manufacturer’s instructions of the EpiTech Bisulfite Kit (QIAGEN, Valencia, CA, USA). Bisulfite-sequencing PCR (BSP) amplification was performed using 1 μl purified bisulfite-conversed DNA (50 ng/μl), 10 μl 2× Prime Taq Premix, 1 μl Upstream Primer (5 pmole/μl), 1 μl Downstream Primer (5 pmole/μl), and 7 μl DEPC-treated water. BSP conditions included initial denaturation at 95 °C for 5 min, denaturation at 95 °C for 30 s, annealing at 53 °C (*ATP a1*), 50 °C (*ATP b1*), 55 °C (*TOR*), 57 °C (*GRF 5*), or 53 °C (*GRF 6*) for 30 s, and extension at 72 °C for 30 s; 40 cycles were conducted, with the final extension at 72 °C for 5 min. Primers for BSP were designed using the MethPrimer (http://www.urogene.org/methprimer/) (see Supplementary Table S5). BSP amplification products were purified using the MiniBest Agarose Gel DNA Extraction Kit (TaKaRa Bio Inc., Kusatsu, Shiga, Japan), following the manufacturer’s instructions.

### Cloning and methylation sequencing

Purified BSP amplification products were ligated to the pGEM-T Easy Vector and transformed into competent JM109 cells by using the pGEM-T Easy Vector system I (Promega, Madison, WI, USA). White colonies were selected from the ampicillin/X-Gal/IPTG plates and colony PCR was performed using vector-directed primers to confirm the presence of inserts based on their expected fragment size. Ten positive colonies from each plate were inoculated into LB medium with ampicillin for plasmid isolation. Plasmids containing the target DNA were extracted and purified by using the Plasmid Midi Kit (QIAGEN) and sequenced by using the ABI Prism BioDye Terminator version 3.1 sequencing system. Cytosine methylation results were analyzed using the CyMATE online methylation sequence analysis software^48^. Average methylation level expressed as percentage (%) per site for each of the three types of cytosines, CG, CHG and CHH, were calculated by dividing the number of non-converted (methylated) cytosines by the total number of cytosines of each type within the sequenced region.

### Statistical analysis

All data are presented as the mean value ± standard error of three replicates. Statistical analyses were performed using the Statistical Package for the Social Sciences (SPSS version 16.0; SPSS, Chicago, IL, USA). Data were analyzed by one-way ANOVA and Fisher’s least significant difference (LSD) test to determine treatment differences.

## Additional Information

**How to cite this article**: Zhang, J. J. *et al*. Growth-inducing effects of argon plasma on soybean sprouts via the regulation of demethylation levels of energy metabolism-related genes. *Sci. Rep.* **7**, 41917; doi: 10.1038/srep41917 (2017).

**Publisher's note:** Springer Nature remains neutral with regard to jurisdictional claims in published maps and institutional affiliations.

## Supplementary Material

Supplementary Information

## Figures and Tables

**Table 1:** Effects of plasma at 22.1 kV for different exposure durations on germination and production rates of soybean sprouts.

Exposure duration	Germination rate (%)	Production rate (%)
0 s	80.41 ± 3.00	4.97 ± 0.65
12 s	92.51 ± 2.08**	8.31 ± 0.75**
24 s	91.41 ± 2.85**	7.48 ± 0.39**
48 s	89.53 ± 2.09**	7.08 ± 0.47**
1 min	89.38 ± 2.04**	6.73 ±± 0.84**
2 min	75.51 ± 1.08*	4.11 ± 0.58
3 min	70.41 ± 1.87**	3.37 ± 0.27**

Within a column: ^*^p < 0.05 versus control; ^**^p < 0.01 versus control, according to LSD test.
