# Supplementary material for: Growth-inducing effects of argon plasma on soybean sprouts via the regulation of demethylation levels of energy metabolism-related genes
Source: Sci Rep. 2017 Feb 7;7:41917. doi: 10.1038/srep41917 (PMC5294452; doi:10.1038/srep41917)
Supplement: Supplementary Information [file srep41917-s1.pdf]

Growth-inducing effects of argon plasma on soybean sprouts via the regulation of demethylation levels of energy metabolism-related genes

Jiao Jiao Zhang[1, #], Jin Oh Jo[2, #], Do Luong Huynh[1], Raj Kumar Mongre[1], Mrinmoy Ghosh[1], Amit Kumar Singh[1], Sang Baek Lee[2], Young Sun Mok[2, *], Park Hyuk[3], and Dong Kee Jeong[1, *]

[1]Laboratory of Animal Genetic Engineering and Stem Cell Biology, Department of Animal Biotechnology and Advance Next Generation Convergence Technology, Jeju National University, Jeju 690756, South Korea

[2]Department of Chemical and Biological Engineering, Jeju National University, Jeju 690756, South Korea

[3]Intellectual Property Law Firm PCR, Seoul 06194, South Korea

[*]*Correspondence and requests for materials should be addressed to D. K. J. (dkjeong@jejunu.ac.kr) and Y. S. M. (smokie@jejunu.ac.kr)*

[#]*These authors contributed equally to this work.*

Supplementary Figure S1. Effects of plasma at different potentials for 1 min on (a) 6-day-old soybean sprouts appearance, (b) average stem length, (c) average root length, and (d) average weight of soybean sprouts on days 2, 4, and 6. Error bars indicated standard error (n = 3). *, $p < 0.05$ versus control; **, $p < 0.01$ versus control, according to LSD test.

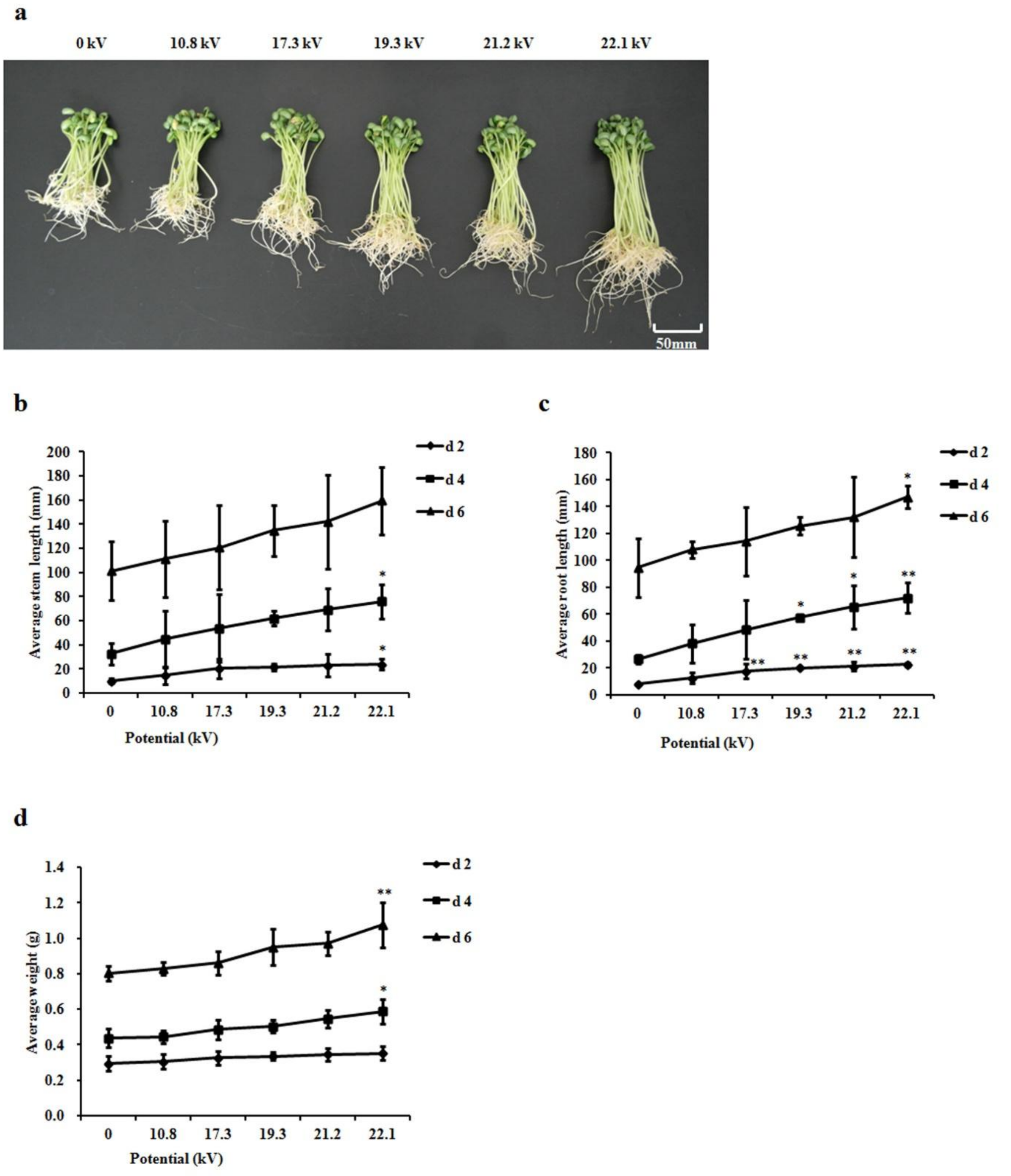

Supplementary Figure S2. Effects of plasma at different potentials for 2 min on (a) 6-day-old soybean sprouts appearance, (b) average stem length, (c) average root length, and (d) average weight of soybean sprouts on days 2, 4, and 6. Error bars indicated standard error (n = 3). *, $p < 0.05$ versus control; **, $p < 0.01$ versus control, according to LSD test.

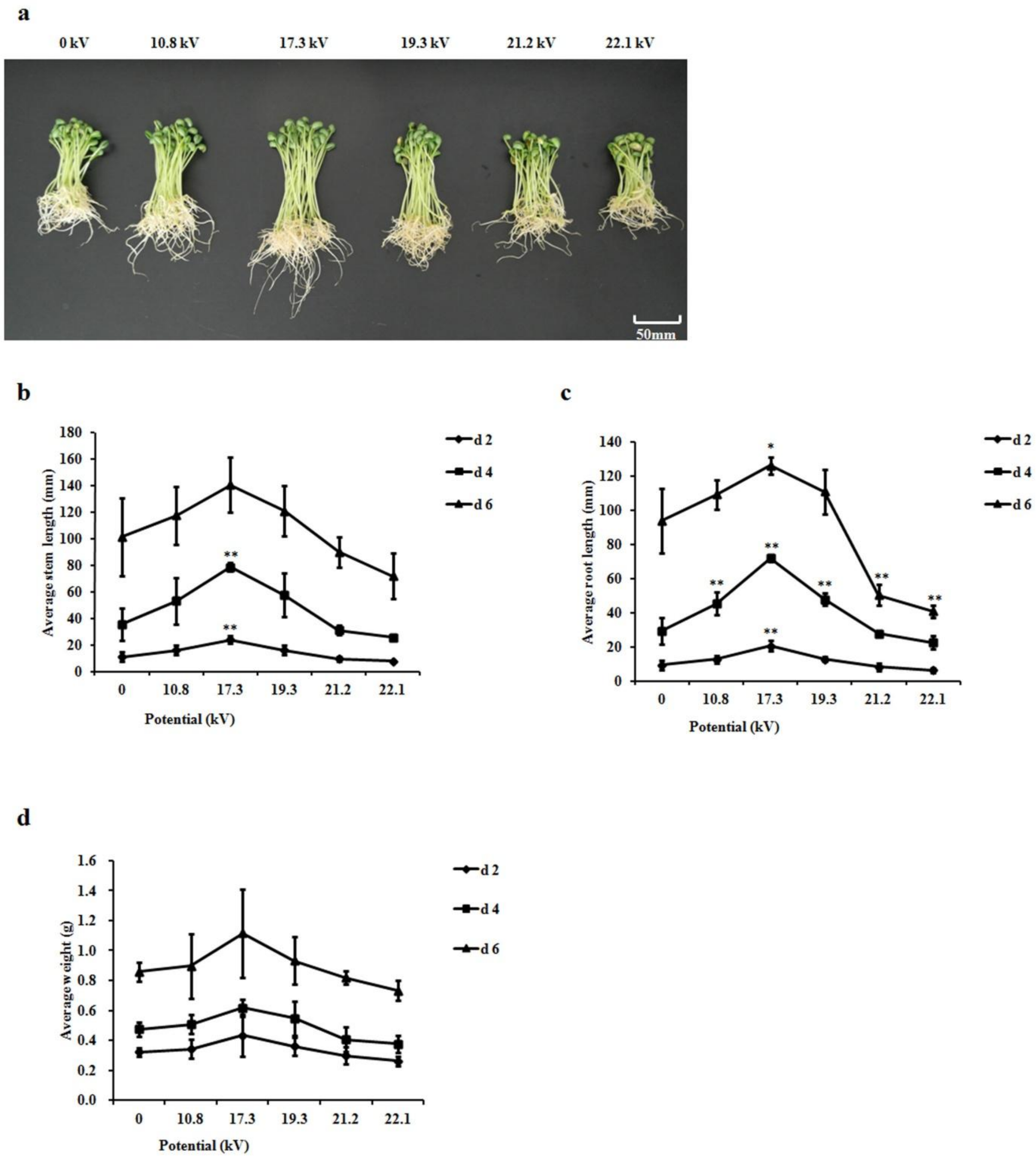

Supplementary Table S3. Effects of plasma at different potentials and exposure durations on germination and production rates of soybean sprouts

Potential (kV)	Germination rate (%)		Production rate (%)	
	Exposure duration		Exposure duration	
	1 min	2 min	1 min	2 min
0	80.44 ± 2.03	81.42 ± 2.77	4.72 ± 0.51	5.11 ± 0.35
10.8	80.42 ± 1.22	86.42 ± 1.94*	4.81 ± 0.24	5.88 ± 0.67
17.3	83.36 ± 3.05	88.33 ± 1.96**	5.28 ± 0.33	7.82 ± 0.41**
19.3	83.55 ± 1.97	84.38 ± 4.20	5.93 ± 0.57*	5.93 ± 0.39*
21.2	86.54 ± 2.20**	78.60 ± 1.03	6.12 ± 0.65*	4.72 ± 0.52
22.1	90.42 ±1.93**	74.60 ± 2.13**	6.80 ± 1.06**	4.13 ± 0.24*

Within a column: *, $p < 0.05$ versus control; **, $p < 0.01$ versus control, according to LSD test.

Supplementary Table S4. Primer sequences for RT-PCR

Gene	Sequence number	Sequence position	Product length (bp)	Sequence (5'to3')
β-actin	CA937380.1	248-377	130	F: CTACTGGGTTTAGAGGCTCATGG<br>R: AACCTGCCATAGAAGAAGTGCC
ATP a1	XM_003540456.2	397-534	138	F: CCTGCTCCCGGTATTATTTCG<br>R: TGCTGTTTTACCCGTTTGTCTG
ATP a2	XM_006588217.1	184-347	164	F: GGCATTGCTTTGAATTTGGAA<br>R: ATTGGTTTAGCCAGGGCATTTA
ATP b1	XM_003536602.2	396-492	97	F: AGGCGTTGTCCGAACCATT<br>R: ACCAACTGGAACGGTAATAGGG
ATP b2	XM_003555884.2	603-706	104	F: TGAGCAAGAAACCGCACAGC<br>R: CAGCACCACCAAACAACCCA
ATP b3	XM_006584177.1	211-399	189	F: GTTGGAGAACGAACCCGAGAA<br>R: AGCATCACGGAAGTGTTTAGCC
ATP MI25	XM_003550048.2	419-545	127	F: CTTCCTGTCGCATCCGTCTTC<br>R: ACCACCAAGCTCTCTCGAATGA
TOR	XM_003517543.2	182-293	112	F: TGGGCAAAATCATCCACAGG<br>R: ATGCTGCCGAACTTTGTCAAC
GRF 1	XM_003534897.2	849-1007	159	F: CCACTCAAAGCAGCAGCAACA<br>R: TCATCCAAATCAAGCCAAGACC
GRF 2	XM_003518751.2	176-316	141	F: TGAACAGACCAAGTGAGAAGCG<br>R: AATCAGCAGTGAAAGCCAACG
GRF 3	XM_003542518.2	743-854	112	F: AGCCTGACAATGGTGAAAGCA<br>R: CCAGTGGACAATTTCAACGACA
GRF 4	XM_003537618.2	609-702	94	F: GGAGCCACAGATGGACAACACT<br>R: ATCACCCTGCAACATGGAATTAT
GRF 5	XM_003547671.2	100-294	195	F: ACAGGAACACCTATCCCACCAG<br>R: TGCCTCCTTTGAACATCTCCAT
GRF 6	XM_003549320.2	662-835	174	F: ACAGCACAGACCCTGAGCCA<br>R: TGTTAGTGGTGGTGAGGGCAT

ATP a1, Glycine max ATP synthase subunit alpha, chloroplastic-like (LOC100815582). ATP a2, Glycine max ATP synthase subunit alpha, chloroplastic-like (LOC102661828). ATP b1, Glycine max ATP synthase subunit beta, mitochondrial-like (LOC100797948). ATP b2, Glycine max ATP synthase subunit beta, mitochondrial-like (LOC100789705). ATP b3, Glycine max ATP synthase subunit beta, mitochondrial-like (LOC100783395). ATP MI25, Glycine max ATP synthase protein MI25-like (LOC100816723). TOR, Glycine max serine/threonine-protein kinase TOR-like (LOC100816558). GRF 1, Glycine max growth-regulating factor

1-like (LOC100814271). GRF 2, Glycine max root meristem growth factor 2-like (LOC100818175). GRF 3, Glycine max growth-regulating factor 3-like (LOC100817197). GRF 4, Glycine max growth-regulating factor 4-like (LOC100817313). GRF 5, Glycine max growth-regulating factor 5-like (LOC100814760). GRF 6, Glycine max growth-regulating factor 6-like (LOC100819396).

Supplementary Table S5. Primer sequences for bisulfite-sequencing PCR

Gene	Sequence number	Sequence position	Product length (bp)	Sequence (5'to3')	Expected No. of CpGs
ATP a1	XM_003540456.2	123-423	301	F: TGAATAGTTGGAAAAAGTTTAAGTT	13
				R: CATACCAATATTAACACTAACAATAC	
ATP b1	XM_003536602.2	42-408	367	F: TTTTTTTTGAAGATGGTTTTA	38
				R: AATAATCCAAAACCTCCAAC	
TOR	XM_003517543.2	324-612	289	F: TTGAAGAAGTATTTAGAGGAAGAAGTT	17
				R: CCCCAACCCTAACAAAATAAC	
GRF 5	XM_003547671.2	1279-1590	312	F: AGGTTAAGTAAAGAGAAAGAAGTTGAGATA	4
				R: AACACAAATCCTACTATAATCCCCTAC	
GRF 6	XM_003549320.2	987-1162	176	F: GGAGGTTTTTATTTGGGATTTTTTA	6
				R: CCTAATTAACCTTCCACAAACTTTCT	
